# Inter- and Intra-Species Variation and Genetic Diversity of Flea Ectoparasites in Hedgehogs (Mammalia, Erinaceidae) Collected in Northern Algeria

**DOI:** 10.3390/insects16040390

**Published:** 2025-04-06

**Authors:** Ourida Chebbah, Karim Souttou, Karim Ouachek, Mohamed Lounis, Sophie Brun, Arezki Izri, Mohammad Akhoundi

**Affiliations:** 1 Laboratory for Exploration and Valorisation of Steppe Ecosystems, Department of Biology, Faculty of Natural and Life Sciences, University of Djelfa, P.O. Box 3117, Djelfa 17000, Algeria; 2 Parasitology-Mycology Department, Avicenne Hospital, AP-HP, Bobigny, Sorbonne Paris Nord University, 93430 Villetaneuse, France; 3 Unité des Virus Émergents (UVE: Aix-Marseille Université, Università di Corsica, IRD 190, Inserm 1207, IRBA), 13005 Marseille, France

**Keywords:** hedgehog, *Atelerix algirus*, *Archaeopsylla erinacei*, *Ctenocephalides felis*, genetic diversity, Algeria

## Abstract

Hedgehogs are small omnivorous mammals prevalent across Europe, Asia, and Africa. The expanding intrusion of humans into hedgehog habitats and rising popularity of keeping exotic animals like hedgehogs as pets have disrupted the delicate balance of the human–animal–environment interface. Despite their ecological importance, hedgehog-borne flea species have received limited research attention, with most studies focusing on their vectorial role, and except one report, nothing on the species composition and genetic diversity of hedgehog flea specimens. An inventory and phylogenetic investigation, conducted for the first time in Algeria as well as in North Africa, allowed for collecting 45 hedgehogs (15 males and 30 females) and 303 fleas, and analyzing them through morphological and molecular approaches. The hedgehog specimens were subjected to macro- and microscopic examinations based on diagnostic morphological criteria and morphometric measurements, resulting in their identification as *Atelerix algirus.* This study represents the first report of this species’ prevalence in Djelfa. The ecological and geographical diversity of the studied areas, from arid deserts to humid regions, highlights the adaptability and resilience of *A. algirus* to diverse habitats. Additionally, 271 and 32 flea specimens were morphologically identified as *Archaeopsylla erinacei* and *Ctenocephalides felis*, respectively. Molecular examination targeting ITS1-rDNA further confirmed the morphological identification of fleas species. Phylogenetic analysis of fleas’ ITS1-rDNA sequences revealed heterogeneity, with the specimens clustering into two distinct clades. The first clade comprised two populations of *A. erinacei,* while the second clade included *C. felis* specimens of this study, grouped with homologous sequences from various regions worldwide. No correlation between flea species and geographical locations was observed demonstrating the sympatric distribution of flea specimens in the studied regions. In addition, no hybrid or genetic combination notion was observed among the flea specimens processed in this study compared with those coming from other countries. These findings contribute to our understanding of the species composition and distribution of hedgehogs and their flea ectoparasites in Algeria and provide a baseline for future epidemiological and entomological research in the country.

## Simple Summary

In Algeria, hedgehogs are abundant in diverse habitats, ranging from rural grasslands to urban gardens. However, research on the fleas that infest hedgehogs in this country has been limited to sporadic investigations focusing mainly on their role as vectors of pathogens. This study investigates flea ectoparasites of hedgehogs in northern Algeria, focusing on species composition, genetic diversity, and phylogeny. A total of 303 fleas were collected from 45 hedgehogs (*Atelerix algirus*) across 4 regions. Morphological and molecular analyses identified two flea species: *Archaeopsylla erinacei* (271 specimens) and *Ctenocephalides felis* (32 specimens). Phylogenetic analysis of ITS1-rDNA sequences revealed two distinct clades, reflecting genetic variation among populations. These findings provide valuable insights into the hedgehog-associated fleas, their distribution, genetic diversity, and phylogenetic relationships of flea species infesting hedgehogs in northern Algeria, and are critical for identifying zoonotic risks and developing effective control measures.

## 1. Introduction

Hedgehogs, small omnivorous nocturnal synanthropic mammals, have a worldwide distribution, prevalent in various temperate and tropical regions of Europe, Asia and Africa, as well as in New Zealand imported by humans [1,2,3]. They are commonly found in terrestrial habitats including forests and cultivated areas and contribute significantly to insect population control, serve as prey for larger predators, and play a role in seed dispersal [4]. They belong to the Erinaceidae family, with five genera (*Erinaceus*, *Atelerix*, *Hemiechinus*, *Paraechinus* and *Mesechinus*) and seventeen species [1,5,6]. Among them, six species of *Atelerix algirus*, *Erinaceus concolor*, *E. europaeus*, *E. roumanicus*, *Hemiechinus auratus* and *Paraechinus aethiopicus* are prevalent in the Mediterranean basin [7].

Human encroachment into natural habitats has increased interactions between humans, wildlife, and domestic animals, particularly in urban and peri-urban environments. Hedgehogs, common in such environments, serve as reservoirs of VBPs (vector-borne pathogens) and a host of arthropod vectors, facilitating zoonotic transmission. They may harbor pathogenic agents like *Anaplasma phagocytophilum* or *Borrelia burgdorferi* sensu lato [3] and carry a variety of bacteria and protozoa (e.g., *Salmonella* spp., *Leptospira* spp., *Giardia* spp., and *Cryptosporidium* spp.) through their ectoparasites [8,9].

Hedgehogs are often infested by various ectoparasites, particularly fleas. Adult fleas are small, 1.0 to 3.3 mm, brownish, holometabolous blood-sucking insects with long hind legs adapted for jumping [10]. They belong to the Siphonaptera order, currently comprising 249 genera and 2215 described taxa [11]. Some species such as *A. erinacei* display host-specific behavior by infesting hedgehogs. Conversely, some Pulicidae species such as *C. felis* or *Pulex irritans* exhibit a high degree of promiscuity, being found across a wide range of carnivores [12].

In Algeria, hedgehogs are common in various environments, often sharing habitats with humans and domestic animals, increasing the risk of parasite spillover. Based on an investigation carried out by Kowalski and Rzebik-Kowalska [13], two main species are recognized:

(i) *Atelerix algirus* Lereboullet, 1842: Native species, adapted to arid and semi-arid regions of North Africa, including Algeria. It is mostly found in the Mediterranean belt along the north of the country, with the southernmost records in the literature being from Ain Sefra, Ain El Orak, Laghouat and Biskra [2,13,14]; and (ii) *Paraechinus aethiopicus* Ehrenberg, 1832: Inhabits a sub-desert band between the Saharan Atlas and the true desert, roughly delimited by Beni Abbes to the west, Ain Sefra to the north, Biskra to the east, and El Golea in the central part of the country. Further south, it is also present in the mountains of the Central Sahara, including the Adrar Ahnet and the Hoggar. These species coexist in Algeria’s Hauts Plateaux, displaying low genetic diversity and a parapatric distribution, where their ranges meet but do not significantly overlap [13,15,16,17].

Fleas are known vectors of several diseases such as typhus, bubonic plague, tularemia, protozoan and helminthic infections worldwide [18,19]. Furthermore, they cause a nuisance and discomfort to their host, resulting in local itchy reactions [20]. The research into the ectoparasitic fauna of hedgehogs in the world has identified flea species including the following:

(i). *Archaeopsylla erinacei* (known as the hedgehog flea) is the most prevalent species infesting hedgehogs worldwide [21,22]. It is frequently reported in both wild and urban settings where hedgehogs are present. Its host-specific behavior and adaptation to hedgehog hosts makes it a potential vector of zoonotic pathogens such as *Rickettsia* spp. and *Bartonella henselae* [23]. Despite its primary association with hedgehogs, it also infests other animals including cats [24], dogs [25], foxes [26] and occasionally humans [27].
(ii). *Ctenocephalus felis*, a versatile species, infests a broad range of hosts including fox, rats, dogs, cats, poultry, ferrets, sheep and humans [28,29]. Although primarily a parasite of cats, *C. felis* is a generalist species and has been found on hedgehogs in urban and peri-urban environments. Its presence on hedgehogs may indicate close contact with domestic animals. Consequently, it is of veterinary and economic importance.
(iii). *Ctenocephalides canis* has also been identified on hedgehogs, despite its primarily association with canines, indicating possible interactions between wildlife and domestic animals [30,31].
(iv). *Pulex irritans* is another ectoparasite occasionally found on hedgehogs. This flea has a broad host range and poses a zoonotic risk. Its zoonotic implications are particularly concerning, given its association with the transmission of diseases such as plague (*Yersinia pestis*) and other bacterial infections [32].
(v). *Xenopsylla cheopis* (known as the oriental rat flea) is another hedgehog flea ectoparasite which is common in arid and semi-arid regions, suggesting a broader host range and potential for disease transmission [31].

In Algeria, studying flea ectoparasites on hedgehogs is essential for understanding the ecological interactions between these hosts and their parasites. Additionally, it highlights the potential zoonotic risks associated with these fleas. Despite the potential role of hedgehogs as hosts for several flea species, often residing in urban and suburban areas in proximity to humans as predomestic reservoirs or pets, and the increasing risk of pathogen transmission to humans and domestic animals, there is a scarcity of studies focusing on hedgehog fleas. Particularly, there is very limited research on hedgehogs and their flea ectoparasites, their morphological characteristics, geographical distribution, genetic diversity, as well as the evolutionary relationships among various species and subspecies. Understanding the interactions between hedgehogs, their ectoparasites, and zoonotic pathogens is crucial for developing effective public health strategies and preventing potential outbreaks. Therefore, the main objective of this study was to conduct a morphological and molecular characterization of flea ectoparasites and their populations’ genetics isolated from hedgehogs originating from various geographical regions of Algeria.

## 2. Materials and Methods

### 2.1. Study Areas and Sample Collection

An inventory study was conducted from May 2022 to November 2023 in four districts (zones) located in the central and northern regions of Algeria. These districts comprised the following:

(i). Tizi-Ouzou: This is situated in northern Algeria, 88 km from the capital. The climate is subhumid Mediterranean, with annual precipitation ranging from 600 to 1000 mm. The region is predominantly mountainous, with the Djurdjura massif and the Sébaou valley. The climate varies according to altitude, featuring cold and wet winters and hot and dry summers.
(ii). Boumerdes: A coastal region located in the north of the country, 45 km from Algiers, it possesses a temperate Mediterranean climate with hot and dry summers according to the Köppen–Geiger classification. The average temperature in Boumerdas is 17.5 °C throughout the year with an average precipitation of 645.2 mm.
(iii). Djelfa: This is situated in the central part of Algeria, 300 km south of Algiers. Its climate is characterized by semi-aridity featuring cold winters and hot and dry summers. Precipitation varies from 350 mm in the north to less than 243 mm in the south.
(iv). Tiaret: This is located 300 km southwest of Algiers. It has a continental climate with dry and harsh winters and hot and dry summers, with an average summer temperature of 40 °C. In normal times, it receives 300 to 400 mm of rain per year.

The hedgehogs were captured alive using wire-mesh traps in various locations such as roadsides, forests, gardens and grazing areas. Subsequently, the hedgehogs were anesthetized using isoflurane and transferred to the laboratory, where they underwent careful examinations for ectoparasites harbored, particularly fleas. For this purpose, the fleas were manually removed using forceps and promptly deposited into labeled sterile microtubes containing 70% ethanol. Both the hedgehogs and their associated fleas were then transferred to the Parasitology-Mycology Department of Avicenne Hospital for further morphological and molecular identifications.

### 2.2. Morphological and Molecular Identification of Hedgehogs’ Flea Species

The collected hedgehog specimens were identified at the species level according to diagnostic morphological criteria such as weight, body and head lengths, tail length, hind leg length and ear length. These measurements were conducted under a stereomicroscope (Nikon SMZ745T, Tokyo, Japan) utilizing the identification keys of Corbet [5], Bretagnolle and Attie [33] and Ulutürk and Coskun [34]. At the end of the study and after ectoparasite removal of hedgehogs, they were released in the same locations where captured. Likewise, the adult flea specimens were mounted in Eukitt medium (composed of 5% acrylic resin and 55% xylene) (ORSAtec GMBH, Bobingen, Germany) and identified based on species-specific morphological criteria such as the presence of conical spines on the head, number of spines in pronotal ctenidium, and conical spine at the posterior end of the antennal fossa using the identification keys of Smit [35] and Beaucournu [36].

### 2.3. DNA Extraction, PCR Amplification, and Species Assignation

The fleas’ DNA was individually extracted using Chelex 10% (Bio-Rad, Hercules, CA, USA), and its concentration was assessed with the Qubit DNA broad-range quantification kit (Thermo Ficher, Waltham, MA, USA). The maceration of flea specimens was performed using mechanical, chemical, and thermal lysis with a tissue lyser machine, proteinase K, and a water bath (bain-marie). Subsequently, the extracted specimens underwent conventional PCR amplifications targeting the ITS1-rDNA region. Each PCR reaction was conducted in a final volume of 25 μL, comprising 12.5 μL master mixture (PCR AmpliTaq Gold 360, Applied Biosystem, Carlsbad, CA, USA), 8 μL distilled water, 1 μM each of the forward (ITS1F: 5′-TCATAAGCTCGCGTTGATT-3′) and reverse (ITS1R: 5′-AGCTGGCTGCGTTCTTCAT-3′) primer [21], and 2.5 μL of the extracted template DNA. PCR reactions followed these conditions: initial denaturation at 95 °C for 5 min, followed by 35 cycles at 95 °C for 45 s, annealing at 57.5 °C for 1 min, extension at 72 °C for 1 min 30 s, and a final extension at 72 °C for 4 min. A couple of negative (distilled sterile water) and positive (flea specimen’s DNA previously positive in PCR examination) controls were used for each PCR batch. The amplicons were then subjected to electrophoresis on a 1.5% agarose gel containing ethidium bromide, and the PCR products of successful amplicons were sequenced for species identity’s confirmation and phylogenetic analysis.

### 2.4. Genetic Diversity and Phylogenetic Reconstruction

The amplified DNA amplicons were bidirectionally sequenced in the laboratory using the same primers employed for PCR amplification. Subsequently, the obtained sequences were edited, aligned, and analyzed using Staden package and BioEdit v7.0.0 software [37,38]. Fleas’ identification was performed at the species level utilizing the Basic Local Alignment Search Tool (BLAST) (www.ncbi.nlm.nih.gov/BLAST (accessed on 24 June 2021)). Phylogenetic analysis was conducted using MEGA v.11 software [39]. The inferred phylogenetic tree of flea species identified in this study, along with homologous sequences from GenBank, was constructed using the Maximum Likelihood (ML) method under the Tamura-Nei model. Bootstrap support values were determined through 1000 replicates. The best-fit substitution model was determined using MEGA, and the Nearest-Neighbor-Interchange (NNI) algorithm was employed as the ML Heuristic Method. Furthermore, to visualize the genetic relationships within flea populations, the median-joining algorithms were implemented using NETWORK v. 5 software [40].

## 3. Results

**Figure 1:**
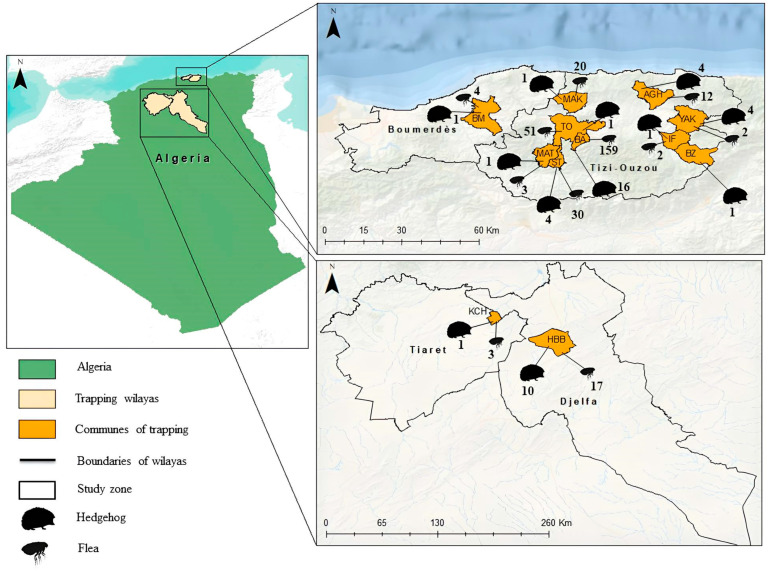
Geographical topology of processed locations for hedgehog and associated flea sampling in northern Algeria. BM: Bordj Menaiel, MAK: Makouda, TO: Tizi-ouzu, MAT: Maatkas, ST: Souk El Thenine, BA: Beni aissi, AGH: Aghrib, YAK: Yakouren, IF: Ifigha, BZ: Bouzeguene, KCH: Kser Chellala, and HBB: Hassi Bahbah.

**Figure 2:**
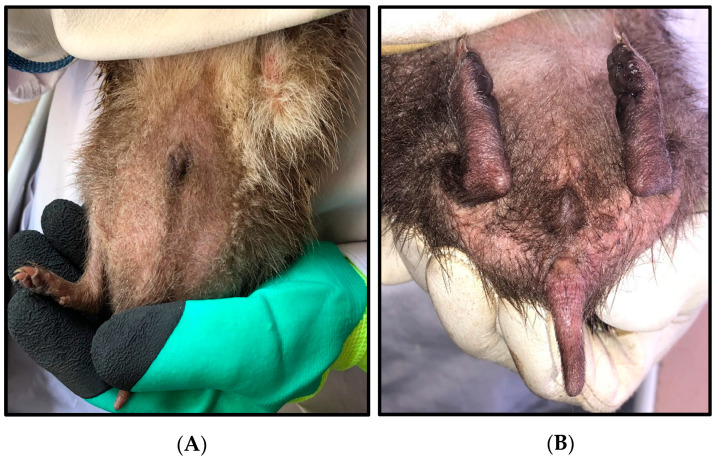
Male (**A**) and female (**B**) *Atelerix algirus* with morphological traits of taxonomical importance, collected in northern Algeria.

The hedgehog collection was conducted in four districts across twelve sites, with the majority of collections taking place in rural areas, located in the central and northern regions of Algeria (Figure 1). A total of 45 hedgehogs were collected, comprising 15 males and 30 females. They underwent macroscopic and microscopic examinations based on diagnostic morphological criteria and morphometric measurements, which identified them as *Atelerix algirus* (Figure 2). Detailed information of morphometric measurements of the hedgehog specimens is given in Table S1.

On the other hand, the hedgehogs were examined for ectoparasitic infestation, revealing that 43 out of 45 (95.5%) were infested by ectoparasites, with 31 out of 43 (72%) exclusively infested by fleas. Two uninfested hedgehogs were male specimens that belonged to the Tizi Ouzou and Djelfa regions (Figure 1, Table 1).

**Figure 3:**
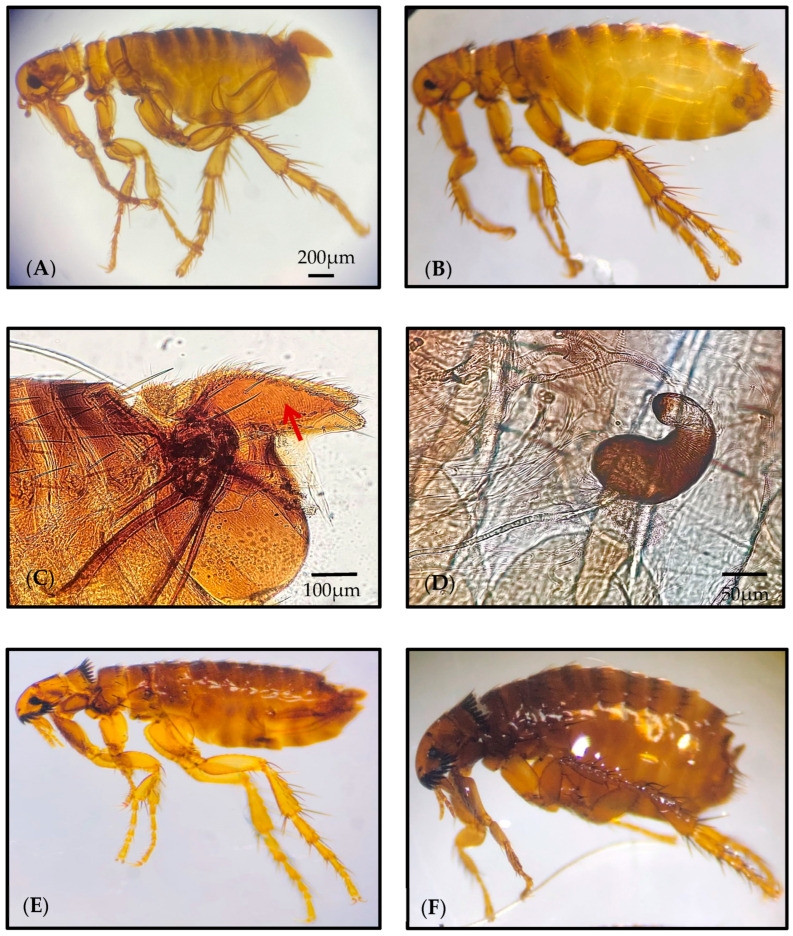
Microscopic views of processed male (**A**) and female (**B**) specimens of *Archaeopsylla erinacei maura*, male basimere of *A. e. maura* (**C**), female spermatheca of *A. e. maura* (**D**), male (**E**) and female (**F**) specimens of *Ctenocephalides felis* identified using morphological and molecular approaches in this study.

Additionally, 303 fleas were collected from infested hedgehog specimens. Among them, 271 and 32 flea specimens were morphologically identified as *Archaeopsylla erinacei* and *Ctenocephalides felis*, respectively (Figure 3). All collected fleas were in the adult life stage and comprised 219 females (197 *A. erinacei*/ 22 *C. felis*) and 84 males (74 *A. erinacei*/10 *C. felis*). Based on morphological examination, all *Archaeopsylla erinacei* specimens processed in this study belonged to *Archaeopsylla erinacei maura*. The males had a basimere length ranging from 325 to 450 µm, with an average length of 380 µm. Similarly, the females had a spermatheca length ranging from 160 to 185 µm, with an average of 172.5 µm (Figure 3C,D). The majority of samples were collected from Beni Aissi (159 specimens), followed by the Tizi-Ouzu region (51). Detailed information regarding the hedgehog and flea specimens processed in this study is provided in Table 1.

The morphological identification of flea specimens was further confirmed through molecular approaches targeting the ITS1-rDNA region. BLAST analysis of ITS1-rDNA sequences revealed ≥97% identity with homologous sequences in GenBank.

**Figure 4:**
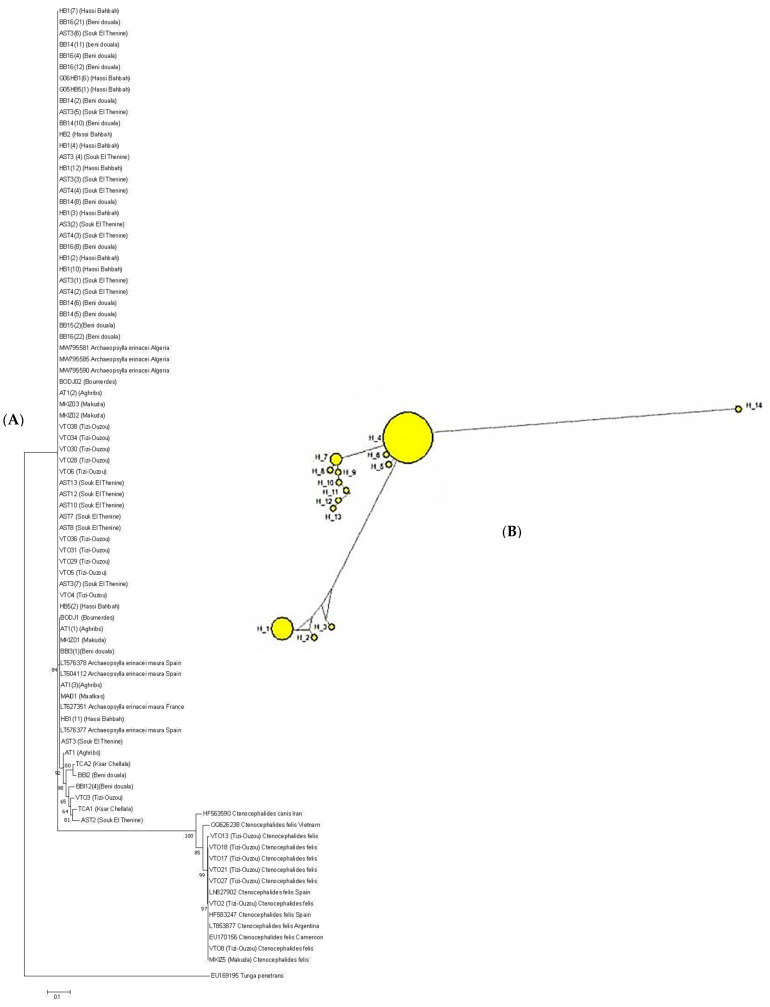
(**A**) Maximum Likelihood (ML) tree reconstructed from ITS1-rDNA sequences of hedgehog-borne flea specimens collected in the present study, accompanied by homologous sequences from GenBank. (**B**) Global analysis median-joining network for various flea populations isolated from processed hedgehogs.

The ML phylogenetic analysis of ITS1-rDNA sequences of flea specimens processed in this study along with homologous sequences from GenBank delineated flea taxa into two major clades. The first major clade consisted of two populations: the first population encompassed flea specimens of this study grouped with homologous sequences from Algeria (e.g., MW795581, MW795590), while the second population included other flea specimens from this study clustered with European sequences from France (LT627351) and Spain (LT604112, LT576377, LT576378). Additionally, the second clade consisted of *C. felis* specimens collected in this study which clustered with counterpart sequences from Iran, Vietnam, Spain, Argentina, and Cameroon (Figure 4A). No hybrid or genetic combination notion was observed among the flea specimens processed in this study compared with those coming from other countries. Moreover, the clustering observed in the median-joining network corresponds closely to the topology of the phylogenetic tree generated by ML analysis (Figure 4B). The median-joining network, incorporating ITS1-rDNA sequences from flea specimens in this study and those from GenBank, illustrates substantial heterogeneity and genetic differentiation among these populations. The distribution of ITS1-rDNA haplotypes within flea species and populations examined in the present study is given in Supplementary Information 2.

## 4. Discussion

In Algeria, a total of three investigations have been conducted on hedgehog fleas in the regions of Bouira, M’sila, Bordj-Bou-Arreridj, Oran and Mascara regions, all focusing on the vectorial role of the collected flea ectoparasites [1,7,41]. *Atelerix algirus* was the principal hedgehog species reported in Bouira, M’sila, and Bordj-Bou-Arreridj followed by *P. aethiopicus* reported only in M’sila [1]. In the present study, we examined 45 hedgehog specimens captured from four distinct districts in northern Algeria—Tizi-Ouzou, Boumerdes, Djelfa, and Tiaret—and identified *A. algirus* as the prevalent species. To the best of our knowledge, this is the first report of this species’ prevalence in Djelfa. Furthermore, our findings confirm the presence of this species in the Tizi-Ouzou, Boumerdes, and Tiaret regions, as previously reported by Kowalski and Rzebik-Kowalska [13] over 30 years ago. The geographical and ecological diversity of the studied areas (detailed in the Section 2), characterized by variations in altitude and precipitation ranging from arid desert regions to humid environments, combined with the collection of *A. algirus*, underscores the remarkable adaptability of this species to a wide range of eco-geological conditions, highlighting its resilience and ability to thrive across contrasting habitats. The presence of *A. algirus* has also been reported in other Mediterranean countries and North African regions, such as Libya, Tunisia and Morocco [30,42,43].

Among hedgehog ectoparasites, fleas are of particular interest due to their role in transmitting zoonotic pathogens. Flea-borne organisms are widely distributed throughout the world in endemic disease foci, where components of the enzootic cycle are present. Despite their importance, hedgehog-borne flea species have been the subject of limited research, primarily focusing on their vectorial role [44,45]. Therefore, knowledge about the morphological and molecular characterization of hedgehog flea species remains limited. According to the literature, Jordan and Rothschild [46] were the first to propose morphological criteria for distinguishing *A. erinacei* as the principal flea ectoparasite of hedgehogs and for differentiating its two subspecies. They proposed identifying males based on the length of the basimere, while for females, identification relied on the presence of one or two lateral bristles on the eighth abdominal tergum and four or five lateral bristles on the seventh abdominal sternum, collectively. In 1990, Beaucournu and Launay [47] validated these criteria, excluding the bristle count on the seventh abdominal sternum, and highlighting reliable identification for males. Research by Zurita et al. [21] confirmed that basimere length is a reliable morphological marker for distinguishing males of *A. e. maura* (Seville) from *A. e. erinacei* (Corsica). Moreover, they demonstrated that the total length of the spermatheca is a useful criterion for differentiating females. Specifically, females from Seville (*A. e. maura*) exhibit spermathecae longer than 120 μm, whereas those from Corsica (*A. e. erinacei*) have spermathecae shorter than 120 μm. These findings were supported by molecular and phylogenetic data, highlighting a distinct geographic distribution pattern [21]. Based on our findings, all morphologically examined *Archaeopsylla erinacei* specimens belonged to *A. e. maura*. The latter was identified according to the basimere and the spermatheca length in male and female specimens, respectively.

In the three investigations mentioned above in Algeria, *A. erinacei* and *X. cheopis* were collected from hedgehogs [1,41]. In the present study, a total of 303 flea specimens were collected. Among them, 271 specimens belonged to *A. erinacei,* while 32 specimens were identified as *C. felis* using morphological and molecular approaches. Among the hedgehog flea species reported in this country, no other flea species were found in the specimens collected from hedgehogs in our study areas. The high prevalence of ectoparasite infestation observed in this study (95.5% of hedgehogs examined) aligns with previous research indicating that hedgehogs are frequent hosts of fleas. The predominance of *A. erinacei* is consistent with its host-specific behavior and adaptation to hedgehogs as primary hosts. However, the detection of *C. felis*, a highly promiscuous species, raises important questions about the ecological dynamics of flea populations in northern Algeria. The presence of *C. felis* on hedgehogs may reflect increased contact between hedgehogs and domestic animals, such as cats and dogs, in urban and suburban environments.

In this study, we exclusively used the ITS1 marker, confirming its high variability and effectiveness in flea species identification. Additionally, it can be complemented by mitochondrial markers such as Cyt b and COI to compare results and ensure consistency. The ML phylogenetic analysis of ITS1-rDNA sequences from flea specimens in this study, alongside sequences from GenBank, revealed two primary clades, reflecting genetic variation among flea populations. The first major clade consisted of two *A. erinacei* sub-populations. This clustering of *A. erinacei* specimens suggests a degree of genetic divergence potentially influenced by geographical and ecological factors. Additionally, the second clade comprised *C. felis* specimens from this study, which clustered with sequences from Iran, Vietnam, Spain, Argentina, and Cameroon (Figure 4). The presence of *C. felis* specimens in a separate clade, alongside sequences from diverse regions, highlights the global distribution and genetic heterogeneity of this species. The sympatric distribution of flea species observed in this study, with no clear correlation between species and geographical location (as all *A. erinacei* specimens belonged to *A. e. maura*), may reflect the mobility of hedgehogs and the adaptability of fleas to diverse environmental conditions. This finding emphasizes the importance of considering ecological and behavioral factors in understanding host–parasite interactions. Furthermore, the clustering observed in the median-joining network confirmed the topology of the ML tree. These results confirm the findings reported by Zurita et al. [21], demonstrating significant heterogeneity among *A. erinacei* populations and a slight genetic division between Algerian and European flea specimens. No evidence of hybridization or genetic recombination was observed among the flea specimens analyzed in this study when compared with those originating from other countries, suggesting a distinct genetic stability or limited interbreeding across populations, which could reflect ecological isolation, host-specific adaptation, or geographical barriers restricting gene flow (Figure 4A,B).

## 5. Conclusions

Despite the ecological and epidemiological significance of hedgehogs, hedgehog-associated ectoparasites remain underexplored in many parts of Algeria. An inventory investigation of hedgehogs and their flea ectoparasites collected from four districts in the north of Algeria, utilizing both morphological and molecular approaches, revealed the exclusive prevalence of the hedgehog species *Atelerix algirus*. Additionally, *A. erinacei* and *C. felis* were identified as the flea species infesting hedgehog specimens with the former as the predominant species. The presence of *C. felis* specimens clustering with sequences from diverse regions emphasizes the widespread distribution and adaptability of this species. It also underscores the potential for parasite spillover between wildlife, domestic animals, and humans, particularly in urban and peri-urban settings. Phylogenetic analysis of the fleas’ ITS1-rDNA sequences revealed heterogeneity, with the flea specimens clustering into two distinct clades. The first clade consisted of three populations of *A. erinacei,* while the second one comprised *C. felis* specimens of this study grouped with homologous sequences from Asia (Iran, Vietnam), Europe (Spain), Africa (Cameroon) and South America (Argentina).

These findings contribute to our understanding of the species composition and geographical dispersion of hedgehogs and their flea ectoparasites in Algeria and could serve as a baseline for future epidemiological and entomological investigations in the country. Additionally, further investigations involving a broader range of hedgehog-parasitizing flea specimens from diverse geographic locations and across different months could deepen our knowledge of the population dynamics of hedgehog fleas and their seasonal variations in Algeria.

## Figures and Tables

**Table 1:** Detailed information of hedgehog (*Atelerix algirus*) and flea specimens collected in this study and analyzed using morphological and molecular approaches.

District	Sampling Location (Rural/Urban)	N° of Caught Hedgehogs		N° of Caught Fleas				Total
		♂	♀	*Archaeopsylla erinacei*		*Ctenocephalides felis*		
				♂	♀	♂	♀	
Aghrib	rural	1	3	2	10	0	0	12
Beni Aissi	rural	6	10	48	110	0	1	159
Bouzeguene	rural	0	1	0	0	0	0	0
Tizi Ouzou	urban	1	0	5	17	10	19	51
Ifigha	rural	0	1	0	2	0	0	2
Mâatkas	rural	1	0	0	3	0	0	3
Makouda	rural	0	1	3	16	0	1	20
Souk El Thenine	rural	2	2	5	25	0	0	30
Yakouren	rural	2	2	0	2	0	0	2
Bourdj-Menaïel	rural	0	1	1	2	0	1	4
Hassi Bahbah	rural	2	8	9	8	0	0	17
Ksar Chellala	urban	0	1	1	2	0	0	3
Total		15	30	74	197	10	22	303

## Data Availability

The original contributions presented in this study are included in the article/Supplementary Materials. Further inquiries can be directed to the corresponding author.

## Supplementary Materials

The following supporting information can be downloaded at: https://www.mdpi.com/article/10.3390/insects16040390/s1: Supplementary Information S1-Table S1: Morphometric measurements of hedgehog specimens collected in this study. Supplementary Information S2: Distribution of ITS1-rDNA haplotypes within flea species and populations examined in the present study.
